# Overexpression of *BcHsfA1* transcription factor from *Brassica campestris* improved heat tolerance of transgenic tobacco

**DOI:** 10.1371/journal.pone.0207277

**Published:** 2018-11-14

**Authors:** Xiangtao Zhu, Yang Wang, Yunhui Liu, Wei Zhou, Bin Yan, Jian Yang, Yafang Shen

**Affiliations:** 1 College of Jiyang, Zhejiang A&F University, Zhuji,China; 2 School of Agriculture and Food Science, Key Laboratory of Agricultural Products Quality Improvement Technology in Zhejiang Province, Zhejiang A&F University, Hangzhou, China; 3 Laboratory of Plant Biotechnology, College of Life and Environment Sciences, Shanghai Normal University, Shanghai,China; 4 Institute of Plant Virology, Ningbo University, Ningbo, China; National Taiwan University, TAIWAN

## Abstract

Heat shock proteins (HSPs) are a type of conserved molecular chaperone. They exist extensively in plants and greatly contribute to their survival under heat stress. The transcriptional regulation factor heat shock factor (HSF) is thought to regulate the expression of *Hsps*. In this study, a novel gene designated *BcHsfA1* was cloned and characterized from *Brassica campestris*. Bioinformatic analysis implied that *BcHsfA1* belongs to the *HsfA* gene family and is most closely related to *HsfA1* from other plants. Constitutive overexpression of *BcHsfA1* significantly improved heat tolerance of tobacco seedlings by affecting physiological and biochemical processes. Moreover, the chlorophyll content of transgenic tobacco plants was significantly increased compared with wild type after heat stress, as were the activities of the important enzymatic antioxidants superoxide dismutase and peroxidase. *BcHsfA1* overexpression also resulted in decreased malondialdehyde content and comparative electrical conductivity and increased soluble sugar content in transgenic tobacco plants than wild-type plants exposed to heat stress. Furthermore, we identified 11 candidate heat response genes that were significantly up-regulated in the transgenic lines exposed to heat stress. Together, these results suggested that *BcHsfA1* is effective in improving heat tolerance of tobacco seedlings, which may be useful in the development of new heat-resisitant *B*. *campestris* strains by genetic engineering.

## Introduction

Changes in the natural environment such as heat, drought, and salinity can cause plant growth retardation and even death [1]. Plants have therefore evolved a series of defense responses to adverse environment, in which the accumulation of heat shock proteins (HSPs) plays an important role in sustaining homeostasis [2]. Heat shock transcription factors (HSFs) have been shown to regulate the expression of HSPs [3] in response to physical and chemical stress, and to co-adjusts other important signaling pathways [4].

HSFs exist extensively in plants, with 21 *Hsfs* in *Arabidopsis thaliana* and 34 in soya. They play a critical role in cell homeostasis under various conditions [4], and can be divided into three categories: *HsfA*, *HsfB* and *HsfC* [4–6]. *HsfA* regulates *Hsp* expression following exposure to abiotic stress, especially heat [5].

Heat and oxidative stress both induce plants to produce HSPs, and the biosynthesis of these stress proteins can be considered an adaptive mechanism in which mitochondrial protection is essential [7]. Similarly, the chloroplast HSP protein protects the chloroplast against damage caused by oxidative stress and heat stress [8]. Heat stress was shown to induce an oxidative stress reaction in *Salmonella typhimurium* causing the up-regulation of many antioxidant enzyme genes [9], while the overexpression of catalase (CAT) and superoxide dismutase (SOD) genes increased thermotolerance in *Saccharomyces cerevisiae* [10]. Furthermore, the heat-shock cis element also contributed partially to the induction of the ascorbate peroxidase (APX) gene under oxidative stress [11].

As a traditional vegetable in many countries worldwide, plants of cabbage family are very sensitive to high temperatures [12], which can severely affect both quality and yield. Therefore, the development of new heat-tolerant cabbage varieties is warranted. In the present study, we cloned a gene designated *BcHsfA1* from young seedlings of the traditional non-heading Chinese cabbage *Brassica campestris* ‘Suzhouqing’, which has comparatively strong heat tolerance [13]. We also identified its biological function. Our findings provide new insights into the breeding of novel cabbage varieties that show resistance to hot temperature by genetic engineering.

## Materials and methods

### Plant materials

Seeds of *B*. *campestris* ‘Suzhouqing’ were germinated for 48 h in darkness at 25°C in pots filled with a moist substrate of peat moss. After germination, the seedlings were incubated in a green house and regularly irrigated with tap water. *Nicotiana tabacum* seeds were sterilized and sown on Murashige and Skoog (MS) medium *in vitro* kept at 4°C for 2 days before germination [14] at 25°C. All the photoperiods were 16 h light /8 h dark.

### DNA sequence analysis

Coding sequences were predicted using the opening reading frame (ORF) finder. The nucleotide sequence and deduced protein amino acid sequence were submitted to NCBI and the latter was analyzed with online ExPASy Proteomics Tools (http://au.expasy.org/tools/); multipe sequence alignments were performed using ClustalX software [15]. A phylogenetic tree was compiled using MEGA5.0 software combined with CLUSTAL W alignments [16].

### Expression pattern of *BcHsfA1* under heat stress

Heat stress was administered by exposing *B*. *campestris* seedlings to 42°C for 0, 0.5, 1, 2, 3, 4 and 5h. Leaves were then harvested for RNA extraction using the TRIzol reagent (Invitrogen, Waltham, MA) and treated with DNaseI (Promega, Madison, WI) according to the manufacturers’ protocols. Purified RNAs were used for real-time quantative (RT-q) PCR analysis. Quantification of gene expression was performed using the comparative *Ct* method [17–18]. Data represented the average of three independent experiments.

### Construction of *BcHsfA1* overexpression vectors

Single-strand cDNAs were synthesized from 5μg of total RNA with an oligo (dT) 17 primer through reverse transcription reactions according to the manufacturer’s protocols (PowerScript^TM^, Clontech, Palo Alto, CA). PCR was performed using the primer pairs BcHsfA1-ORF F and BcHsfA1-ORF R (S1 Table). PCR products was inserted into the cloning vector pMD18-T (TaKaRa, Bio, Shiga, Japan) for sequencing as described before [19–20]. The vectors pMD18T-BcHsfA1 and pCAMBIA2300^+^ were double-digested with *Nco*I and *Bst*E II. After purification, the *gus* gene from pCAMBIA2300^+^ was replaced by the *BcHsfA1* coding region to construct the recombinant pCAMBIA2300^+^-BcHsfA1 (S1 Fig). *BcHsfA1* gene was regulated by the cauliflower mosaic virus (CMV) 35S promoter. The blank vector pCAMBIA2300^*+*^ lacking *BcHsfA1* was used as a control. Each of the above-constructed plasmids and the disarmed *Agrobacterium tumefaciens* strain EHA105 harboring the pCAMBIA2300^+^-BcHsfA1 plasmid were used for plant genetic transformation.

### Plant transformation and PCR assay of transgenic plants

Tobacco transformation was carried out as previously described [21]. Transgenic plants were selected on 1/2 MS medium supplemented with 100 μM kanamycin [21–22]. Genomic DNA was extracted from seedlings of transgenic plants using the modified CTAB method [23]. Prime pairs BcHsfA1-35S-F23 and BcHsfA1-QR were used for PCR detection of the transgenic tobaccos (S1 Table).

### Expression profile analysis of transgenic plants byRT-qPCR

Expression profiles of *BcHsfA1* in the transgenic tobacco were analyzed by RT-qPCR using total RNA extracted from the leaves of transformants and wild-type tobacco plants [20, 24]. The primer pair BcHsfA1-real-F/BcHsfA1-real-Rwas used to examine the transcription of *BcHsfA1*, and NtActin-real-F and NtActin-real-R amplified the reference gene (S1 Table).

### Heat tolerance profile in transgenic tobacco

Transgenic tobaccos and wild-type (WT) seedlings grown in a greenhouse were exposed to 42°C as described previously [25], then leaves were harvested at 0, 1, 2 and 3h to determine physiological and biochemical indexes. Leaves treated with heat stress for 3h were collected to detect the expression profiles of two antioxidative-related genes, *SOD* and peroxidase gene (*POD*), three heat stress defense genes, late embryogenis abundant protein 5 (*LEA5*), and early response to drought 10C and 10D (*ERD10C* and *NtERD10D*), and six heat stress marker genes (*NtHSP17*.*6*, *NtHSP18*.*2*, *NtHSP70*, *NtHSP82*, *NtHSP90* and *NtHSP101*) using RT-qPCR as described above.

The amount of chlorophyll (Chl) was determined according to the method of Wang et al. [26]. Total soluble sugar was extracted from leaves by using 80% hot ethanol and the content was determined as reported by Irigoyen et al. [27–28]. MDA concentrations were measured by the thiobarbituric acid (TBA) reaction according to Wang et al. [29]. For the analysis of POD and SOD enzyme activities, the crude enzyme extract were prepared as reported by Zhang and Kirkham [30]. SOD and POD enzyme activity was measured as described previously [31–32]. Determination of the comparative electrical conductivity was conducted by the deflation method [33].

### Statistical analysis

Each experiment was repeated three times, and standard deviation was reported. Statistical significance was analyzed by the one sample *t* test with SPSS software version 11.5 (SPSS Inc, Chicago, IL).

## Results

### Cloning and sequence analysis of *BcHsfA1*

*BcHsfA1* cDNA (1464bp) was cloned using the homologous primer pairs BcHsfA1-ORF F and BcHsfA1-ORF R. It encoded a protein of 487 amino acids, with a deduced molecular mass of 73.2 kDa and an isoelectric point of 5.54. BLAST search in the GenBank database showed that *BcHsfA1* shared high homology sequences from *B*.*napus* and *B*.*rapa*.

Sequence domain prediction revealed that BcHsfA1 protein contained conserved domains including a DNA-bind domain, an oligomerization domain consisted of hydrophobic repeat regions A/B indispensable for oligomerization, a nuclear localization signal (NLS) recognized by the NLS receptor, an activator motif characterized by aromatic, hydrophobic and acidic amino acid residues associated with transcriptional activation and a leucine-rich nuclear exportsignal (NES) for receptor-mediated nuclear export in complex with the NES receptor [4–6] (S2 Fig).

**Fig 1:**
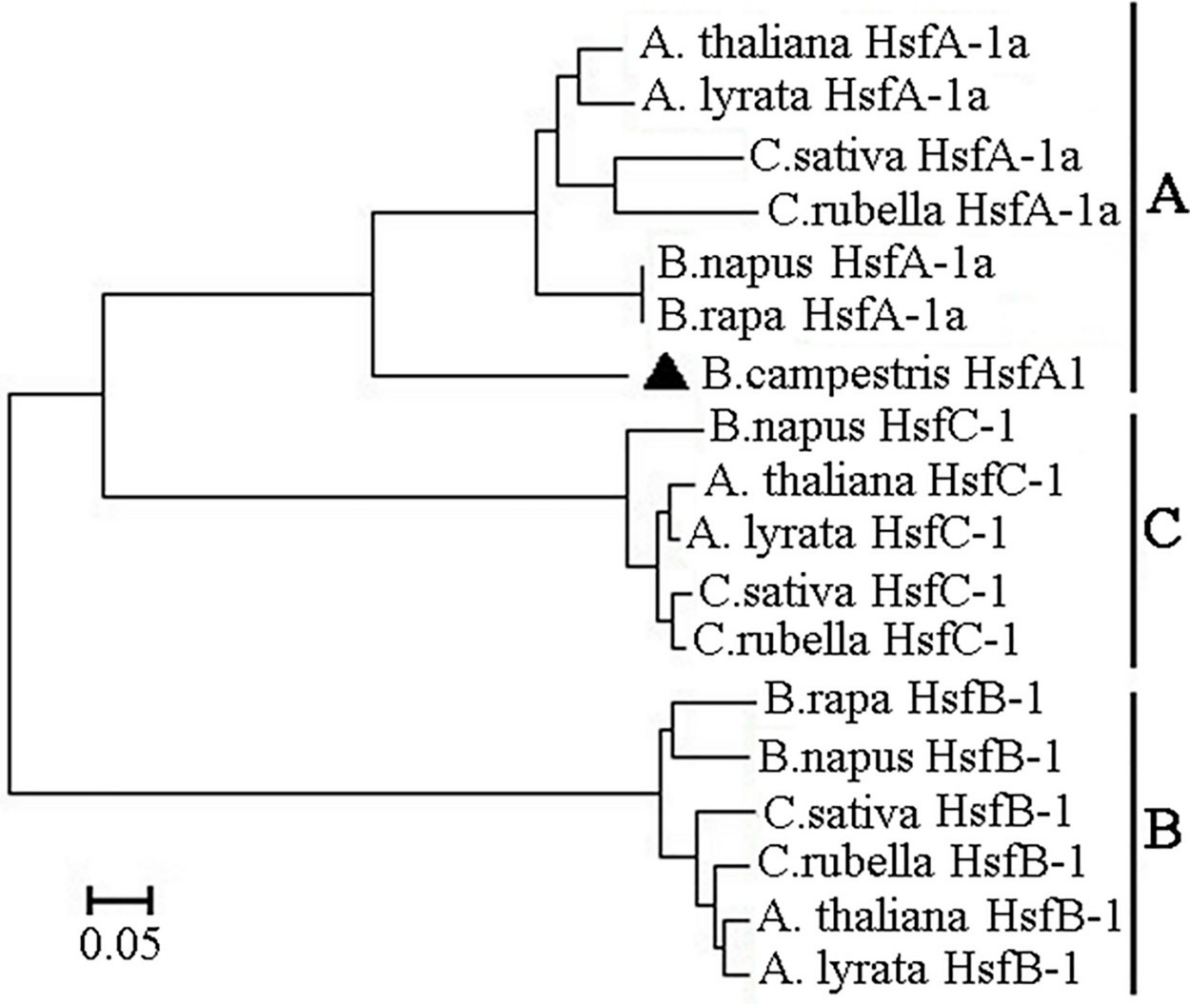
Phylogenetic tree derived from Hsf amino acid sequences. The dendrogram was generated using the neighbor-joining method with MEGA5.0 software. The following Hsf sequences were included: BcHsfA1, *Brassica campestris*; AtHsfA1, *Arabidopsis thaliana*, NCBI amino acid accession number NP_193510.1; AlHsfA1, *Arabidopsis lyrata*, XP_020873200.1; CsHsfA1, *Camelina sativa*, XP_010434623.1; BnHsfA1, *Brassica napus*, XP_013711617.1; CrHsfA1, *Capsella rubella*, XP_006283759.1; BrHsfA1, *Brassica rapa*, XP_009130935.1; AtHsfB1, *Arabidopsis thaliana*, NP_195416.1; AlHsfB1, *Arabidopsis lyrata*, AlXP_020874746.1; CrHsfB1, *Capsella rubella*, XP_006284246.1; BrHsfB1, *Brassica rapa*, XP_009138469.1; CsHsfB1, *Camelina sativa*, XP_010446698.1; BnHsfB1, *Brassica napus*, XP_022550139.1; AtHsfC1, *Arabidopsis thaliana*, NP_189095.1; CsHsfC1, *Camelina sativa*, XP_010466868.1; AlHsfC1, *Arabidopsis lyrata*, XP_020889030.1; CrHsfC1, *Capsella rubella*, XP_006296716.1; BnHsfC1, *Brassica napus*, XP_022562701.1.

A phylogenetic tree was constructed based on the deduced amino acid sequences of BcHsfA1 and other HSF proteins. As showed in Fig 1, HSF proteins were divided into three groups: A, B and C types, and BcHsfA1 was classified into A family.

### *BcHsfA1* responses to heat stress

**Fig 2:**
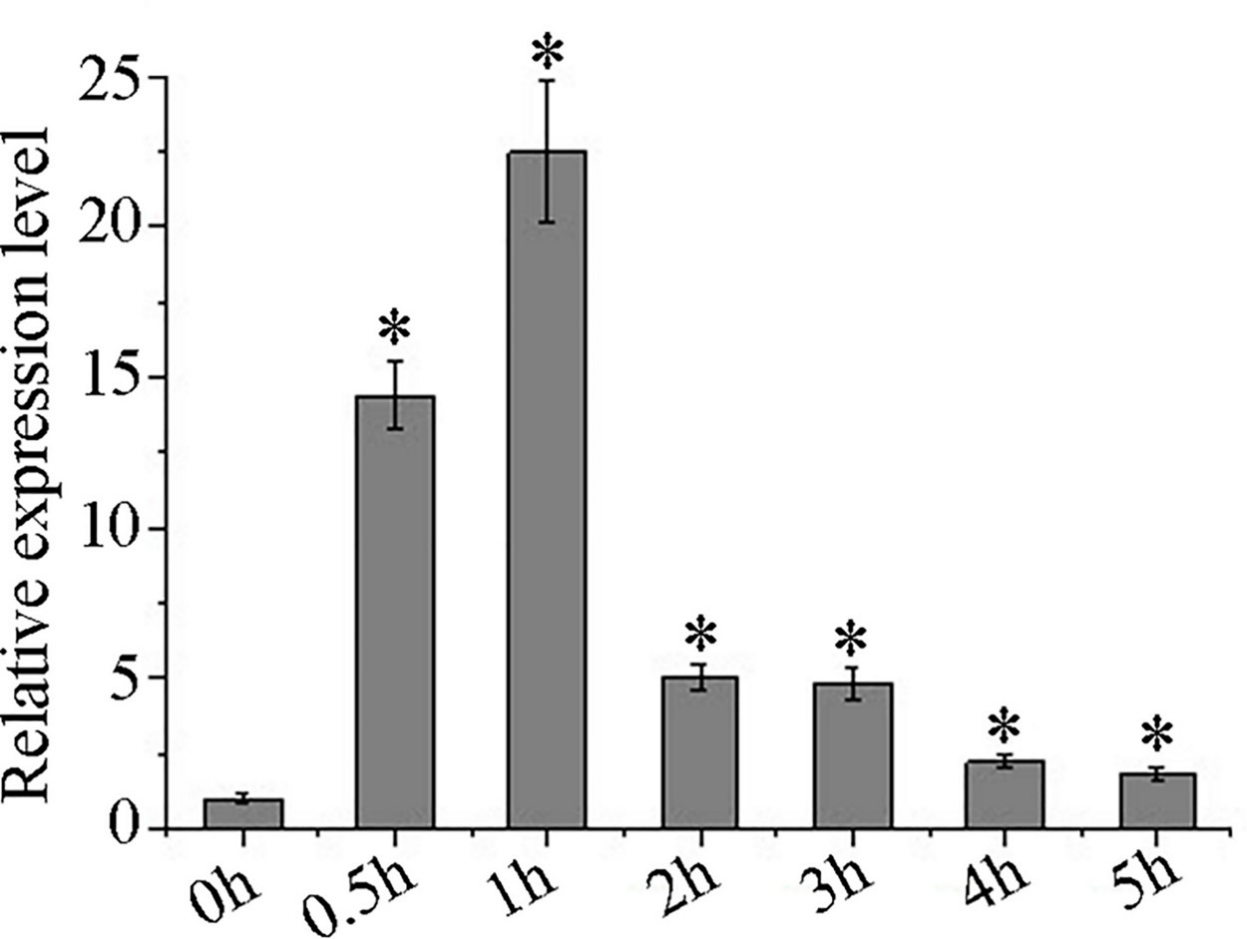
Expression profile of *BcHsfA1* under heat stress. Seedlings of *B*. *campestris* were under heat treatment at 42°C for 0, 0.5, 1, 2, 3, 4 and 5h. The *BcActin* gene was used as an internal control. Error bars indicate standard errors of the mean. Asterisks show significantdifferences based on the *t* test (*p<0.05). The experiment was repeated three times.

To determine the transcription characteristics of *BcHsfA1* exposed to heat stress, seedlings of *B*. *campestris* were incubated at 42°C for heat treatment with 0, 0.5, 1, 2, 3, 4 or 5 h. *BcHsfA1* expression was rapidly triggered at 0.5h and peaked at 1 h with a 22.5-fold increase (Fig 2). The accumulation of *BcHsfA1* then reduced sharply, and stabilized 4h later.

### Overexpression of *BcHsfA1* improved the growth of transgenic plants under heat stress

To study the biological function of *BcHsfA1*, pCAMBIA2300^+^-BcHsfA1 was overexpressed in *N*.*tabacum* to produce T_0_ transgenic tobacco plants. PCR analysis identified a clear band at 1464bp for transgenic but not non-transgenic plants (S3 Fig). This suggested that *BcHsfA1* had been successfully introduced into the transformants.

**Fig 3:**
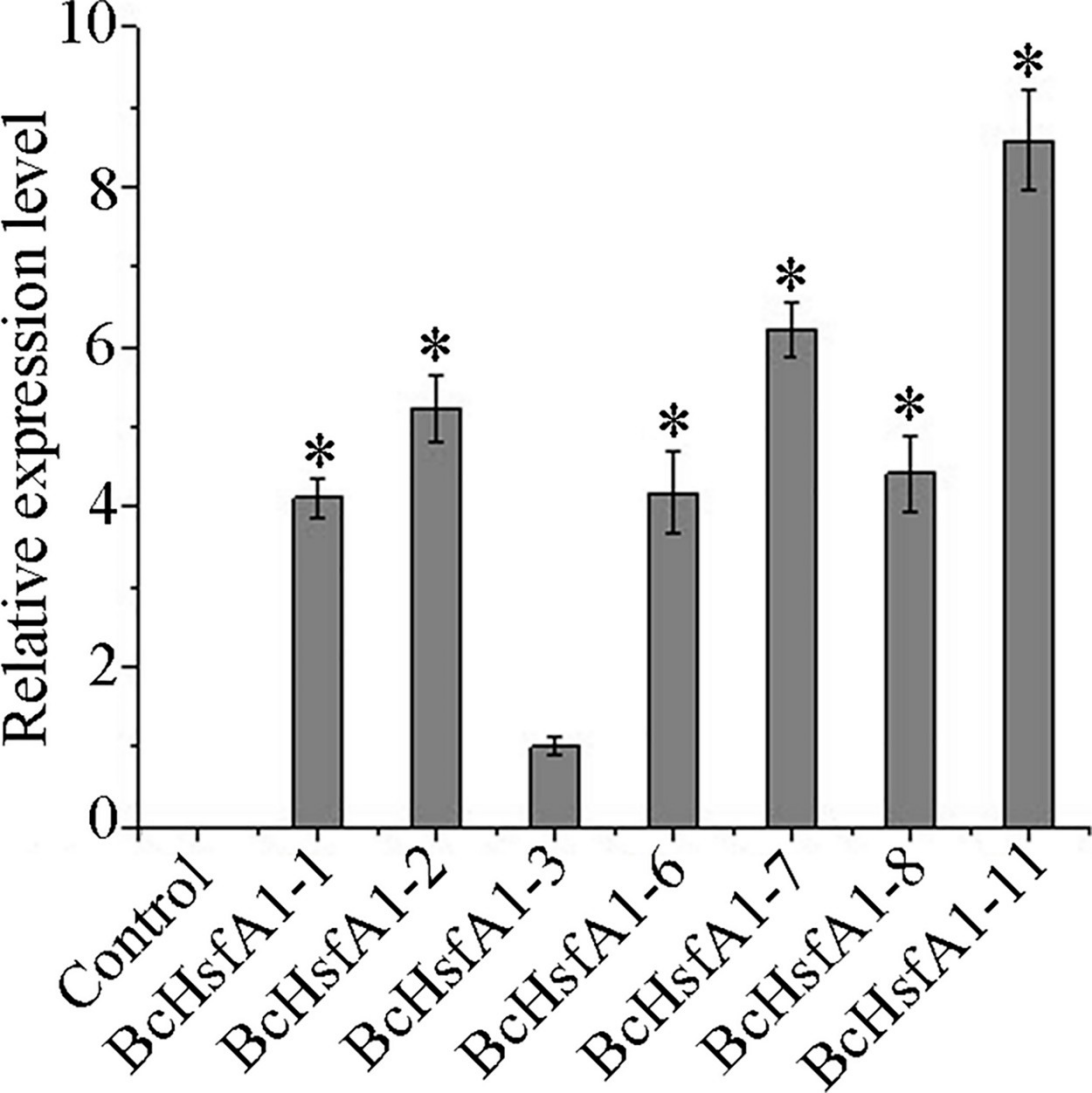
Real-time quantitative PCR analysis of transgenic tobacco. Horizontal line indicates the threshold value. 2^-ΔΔCT^ values (normalized to the endogenous β-actin control) were averaged for each transgenic tobacco (lines 1, 2, 3, 6, 7, 8, and 11). The expression level of line 3 was considered the reference.

RT-qPCR analysis was conducted to determine the transcription of *BcHsfA1* in transgenic plants. Total RNA was extracted from transgenic seedlings (line 1, 2, 3, 6, 7, 8 and 11) and was subjected to RT-qPCR using the transcription level of line 3 as a reference. *BcHsfA1* exhibited a varied expression pattern in different transgenic plants (Fig 3). Line 11 showed the highest *BcHsfA1* transcription, whereas line 3 showed the lowest. Line 1, 2, 6, 7 and 8 showed moderate *BcHsfA1* transcription.

Three transgenetic tabocco lines (2, 7 and 11) showing high *BcHsfA1* expression were selected for subsequent heat stress analysis. Under heat stress (42°C), the transgenic tobacco lines showed more growth and less damage than WT (S4 Fig), suggesting that *BcHsfA1* overexpression improve the heat tolerance of transgenic plants.

### Overexpression of *BcHsfA1* improved the Chl biosynthesis capacity in transgenic plants under heat stress

**Fig 4:**
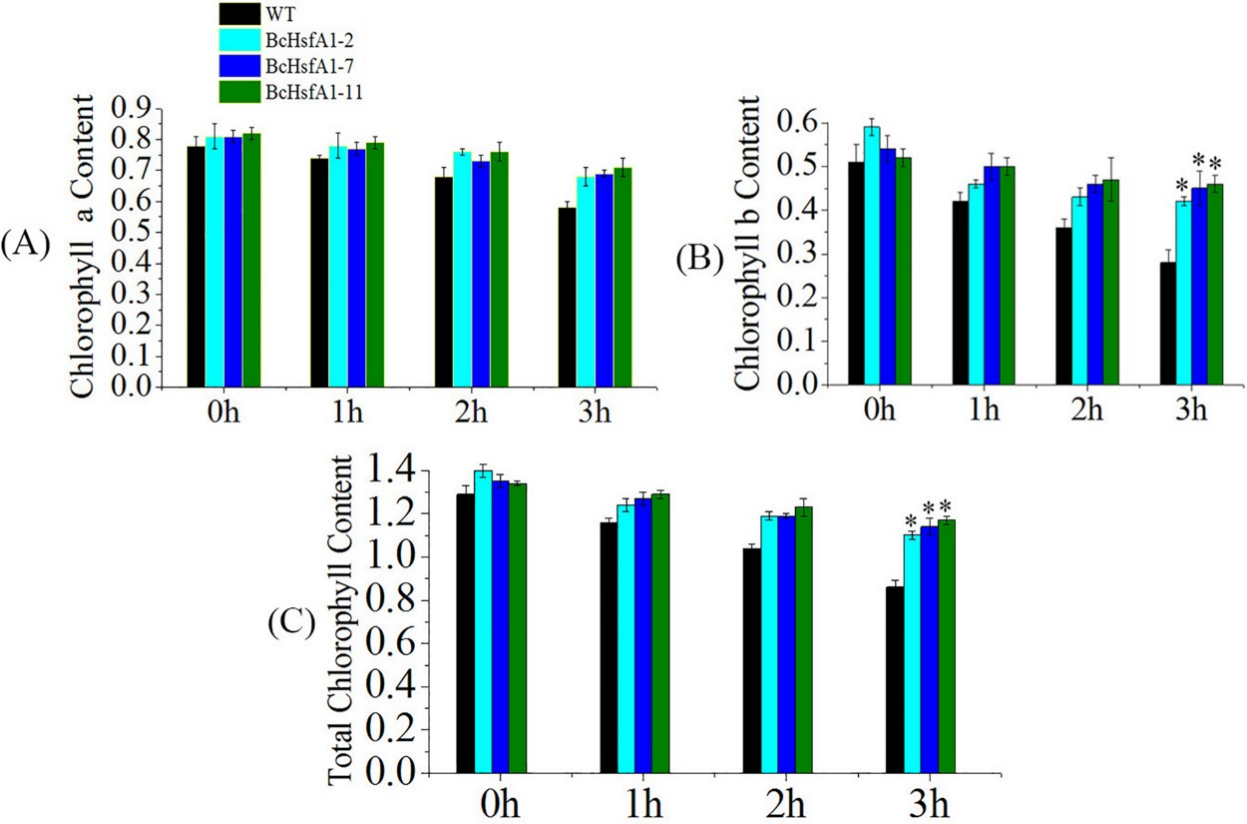
Detection of chlorophyll content inthe leaves of *BcHsfA1* transgenic and WTtobacco under 42°C heat stress. (A) Chl a content; (B) Chl b content; (C) total Chl content.

The plant Chl content is representative of its photosynthetic efficiency. In this study, total Chl, Chl a and Chl b decreased both in *BcHsfA1* transgenic lines and WT under elongated heat stress (42°C) from 0 to 3 h (Fig 4). However, *BcHsfA1* transgenic lines showed higher contents of total Chl, Chl a and Chl b than the controls, and the extent of Chl decrease was slower than in WT.

### Overexpression of *BcHsfA1* increased antioxidative enzyme levels and alleviated cell damage under heat stress

**Fig 5:**
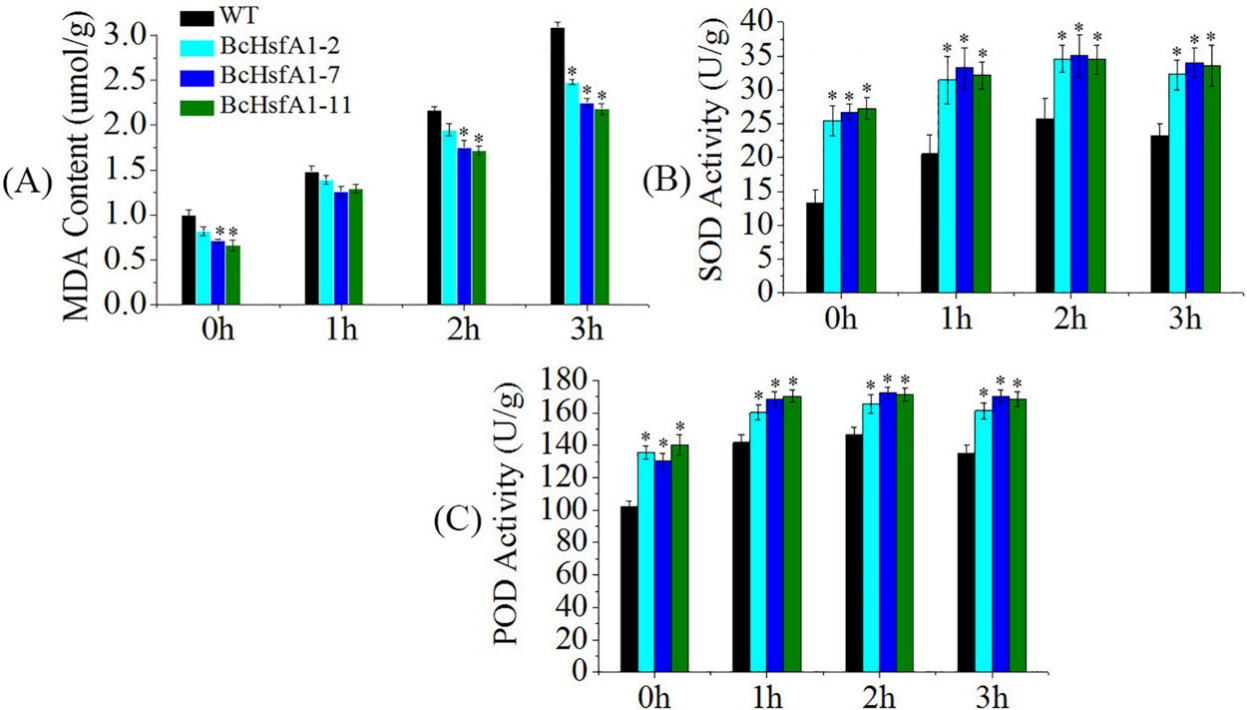
Measurements of MDA content, and SOD and POD activities in leaves of *BcHsfA1* transgenic and control tobacco plantsunder 42°C heat stress. (A) MDA content; (B) SOD activity; (C) POD activity.

WT plants showed higher foundation levels of MDA in their leaves than transformants at 0h under heat stress (Fig 5A). In general, the MDA content increased in leaves of both transgenic and WT plants following heat treatment. However, there was no significant difference between transformants and WT after 1h of treatment (P<0.05). After 2h of heat stress, transgenic line 7 and 11 showed significant lower MDA content than WT, and all three transgenic lines (2, 7 and 11) had significant lower MDA level than WT after 3h of heat stress (P<0.05). This implies that *BcHsfA1* improved the tolerance to heat stress in transgenic tobacco plants.

The MDA content reduction in transgenic plants could be explained by the activation of antioxidant enzymes. Therefore, we next determined the activities of SOD and POD. Transgenic plants showed significantly higher SOD activity than WT at both basal levels (0h) and under heat stress (1, 2, and 3h) (Fig 5B). SOD activity also increased from 0 to 2h heat treatment in both transgenic plants and WT, then declined. All three transformants (line 2, 7 and 11) showed significant higher POD activity than WT from 0 to 3h heat treatment (Fig 5C), and the activity level increased in transformants and WT plants after heat stress although there was no significant difference among 1, 2 and 3h in transgenic plants.These results suggested that *BcHsfA1* overexpression conferred higher SOD and POD activities to protect plants by reducing the lipid peroxidation.

**Fig 6:**
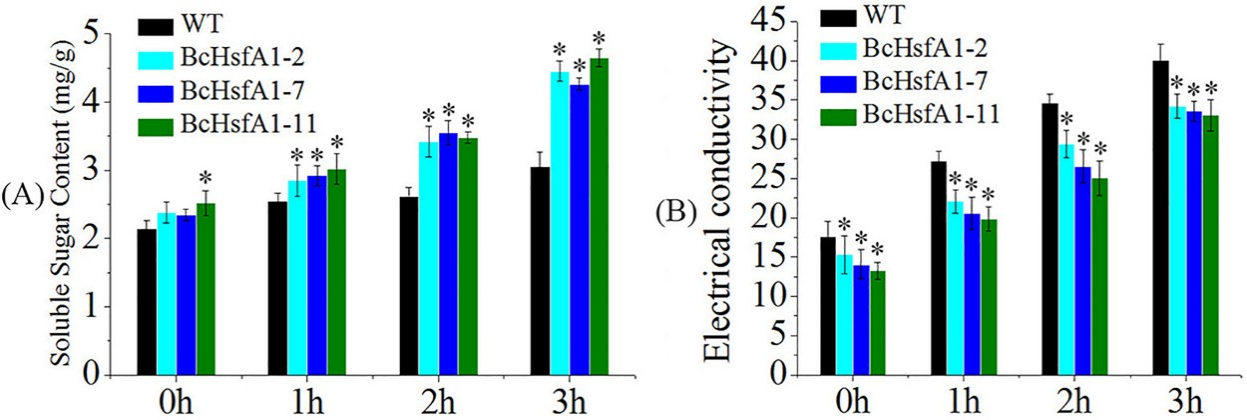
Determination of comparative electrical conductivity and soluble sugar content in leaves of *BcHsfA1* transgenic and control tobacco plants under 42°C heat stress. (A) comparative electrical conductivity. (B) soluble sugar content.

The content of soluble sugar and comparative electrical conductivity are effective indicators of plant heat tolerance. We observed that soluble sugar contents increased significantly in leaves of both WT and transgenic plants from 0 to 3h of heat stress (42 ^o^C) (Fig 6A). Prior to heat stress, only line 11 had a significantly higher soluble sugar content than WT. However, after heat stress (1, 2, and 3h), the soluble sugar content was significantly higher in all transgenic lines than WT. As shown in Fig 6B, the comparative electrical conductivity also increased significantly from 0 to 3h heat treatment in both WT and transformants. Moreover, the comparative electrical conductivity of all transgenic lines (lines 2, 7 and 11) was significantly lower than that of WT both before (0h) and after heat stress (1, 2, and 3h), suggesting that less cell membrane damage occurred in transgenic tobacco plants than WT after heat stress.

### Overexpression of *BcHsfA1* upregulated the expression of heat stress responsive genes

**Fig 7:**
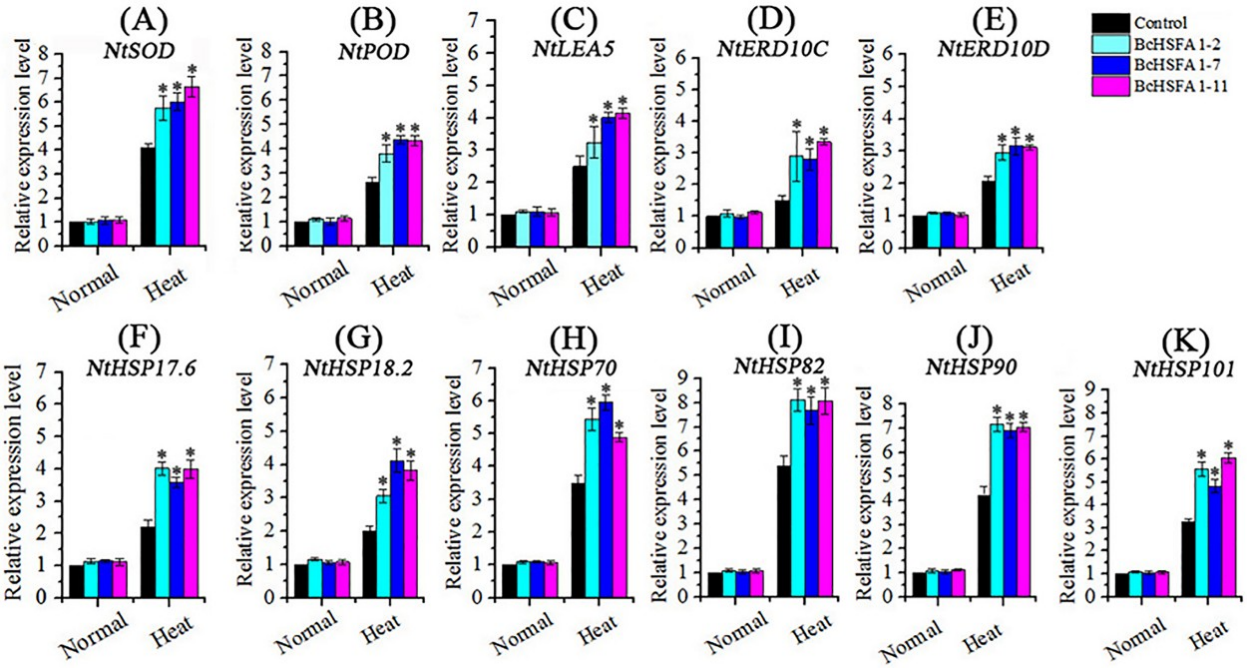
Expression profile of heat-response-related genes. (A) Superoxide dismutase, SOD; (B) Peroxidase, POD; (C) Late embryogenesis abundant protein 5, LEA5; (D) Early response to drought 10C, ERD10C; (E) Early response to drought 10C, ERD10D; (F-K) Heat-shock protein, HSP. Asterisks show significant differences based on the t test (*p<0.05). The experiment was repeated three times.

Next, we used RT-qPCR to examine the expression of antioxidative-related genes including *NtSOD* and *NtPOD*. Heat stress was found to upregulate *NtSOD* and *NtPOD* expression in all tobacco plantstested, but the three transgenic tobacco lines 2, 7, and 11 showed significantly higher (*P*<0.05) *NtSOD* and *NtPOD* expression than WT (Fig 7A and 7B). These expression profiles were consistent with the enzyme activity of SOD and POD in transgenic tobacco lines.

We also measured the expression profiles of three heat stress defense genes (*NtLEA5*, *NtERD10C*, and *NtERD10D*), and six HSP genes (*NtHSP17*.*6*, *NtHSP18*.*2*, *NtHSP70*, *NtHSP82*, *NtHSP90*, and *NtHSP101*). Compared with controls, the expression of all genes was significantly up-regulated in the three transgenic tobacco lines 2, 7, and 11 under heat stress (Fig 7C–7K), indicating that *BcHsfA1* acts as a positive regulator in response to heat stress.

## Discussion

In this study, we cloned and characterized a novel *Hsf* gene from *B*.*campestris*. Sequence analysis revealed that the predicted amino acid sequence of *BcHsfA1* is very similar to that of other plant *HsfA1*s, in particular *AtHsfA1*. The deduced BcHsfA1 protein exhibited motifs and domains that are conserved with HsfA1s from other plants such as *A*. *thaliana* and *B*.*napus* [34–35], suggesting that those domains play important roles in maintaining the HSF function [36–38]. Additionally, BcHsfA1 contained a NLS at its C-terminal [36, 39–40], indicating that it might be located in the nucleus.

The analysis of *BcHsfA1* transcription showed that it responded to heat stress, which is in accordance with earlier findings in other plants [41–42], and implied that *BcHsfA1* may be involved in heat resistance of *B*. *Campestris*. Previous reports also suggested that HSFs could be induced under thermal or other abiotic stresses [43], while *Hsf* overexpression conferred tolerance to heat and other abiotic stresses in plants [3, 37, 42, 44]. To understand the function of *BcHsfA1*, we overexpressed it in tobacco under the control of the 35S CMV promoter and measured various physiological and biochemical indexes.

Environmental stress such as heat and cold damage can affect Chl biosynthesis, resulting in a lower accumulation [45]; therefore, the Chl content can be used as a heat tolerance indicator. In this study, Chl a, Chl b and total Chl was significantly increased in transformants compared with WT after heat stress (Fig 4), suggesting that the higher Chl content might play a vital function in heat tolerance. These results were similar to those of other studiesin wheat, fine fescue and *Arabidopsis* [46–48].

Membranes are the site of primary physiological injury in the plant response to heat stress [49], and damage to membranes causes leakage which induced Chl spillage and degradation in the stoma. Hence, the extent of membrane damage can be evaluated by determining solute leakage [48, 50–51]. In the present study, electrolyte leakage was reduced in transgenic tobacco plants (Fig 6B), which was consistent with previous reports on sweet potato, yeast, *Arabidopsis*, and potato [52–54]. Moreover, the soluble sugar content was also significant higher in transformants than in WT. This could maintain the osmotic pressure of the cell to avoid excessive water loss under heat stress [55].

When plants were exposed to detrimental environmental conditions such as heat, cold, salt, or drought, reactive oxygen species (ROS) including superoxide (O_2_^–^), hydrogen peroxide (H_2_O_2_) and the hydroxyl radical are produced and accumulate [45, 56]. Plants ROS cytotoxicity occurs through enzyme deactivation, the breakage of important cellular components such as cell membranes through oxidative damage, and inhibition of photosynthesis [57]. To prevent this, ROS accumulation is alleviated by inducing activities of ROS-scavenging enzymes [58]. SOD is a key enzyme of the antioxidant defense system, forming the first defense level against superoxide radicals. SOD-catalyzed O_2_^–^ dismutation renders H_2_O_2_ as a reaction product, which in turn is removed by POD, CAT and APX activities [58]. In this study, *BcHsfA1*-overexpressing lines showed much higher SOD and POD activities when exposed to high temperatures (Fig 5). Moreover, as an indicator of membrane peroxidation, the MDA content of transgenic tobacco was lower than that of WT. These increased SOD and POD activities of transformants reflect an active and effective antioxidant response that might be involved in maintaining a lower MDA content, therefore helping plants to cope with the heat stress [59–61].

In conclusion, this study indicated that *BcHsfA1* plays an important role in improving the heat resistance of transgenic tobacco plants. These results will be useful in understanding how plants respond to environmental stresses, and how tolerance to these abiotic stresses can be enhanced by genetic manipulation.

## Supporting information

S1 Fig Schematic of transformation plasmid (*pCAMBIA2300+-BcHsfA1*). (JPG)

S2 Fig Multiple alignment of amino acid sequences for BcHsfA1 and the following other plantHsfA1 proteins. AtHsfA-1a (*Arabidopsis thaliana*, NCBI amino acid accession number NP_193510.1), AlHsfA-1a (*Arabidopsis lyrata*, XP_020873200.1), *CsHsfA-1a* (*Camelina sativa*, XP_010434623.1), BnHsfA-1a (*Brassica napus*, XP_013711617.1). DBD, DNA-binding domain; HR-A and HR-B, hydrophobic repeat regions A/B; NLS, nuclear localization signal; AHA, activator peptide motifs; NES, nuclear export signal.
(JPG)

S3 Fig Representative PCR analysis for the presence of the BcHsfA1 in the transgenic tobaccos. M, size marker; N, untransformed plant (negative control, N); P, p2300^+^-BcHsfA1 (positive control, P).
(JPG)

S4 Fig Phenotype of transgenic and control tobacco plants under heat stress (42°C for 3h). (JPG)

S1 Table Primers used in this study.
(DOCX)
