# Supplementary figures and images for: Overexpression of BcHsfA1 transcription factor from Brassica campestris improved heat tolerance of transgenic tobacco
Source: PLoS One. 2018 Nov 14;13(11):e0207277. doi: 10.1371/journal.pone.0207277 (PMC6235349; doi:10.1371/journal.pone.0207277)

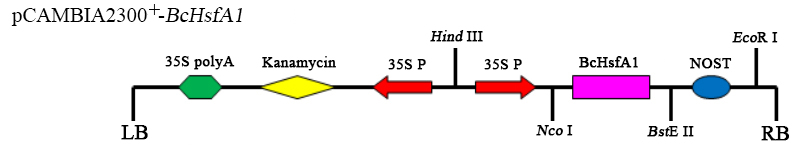

Supplement: S1 Fig — (JPG) [file pone.0207277.s001.jpg]

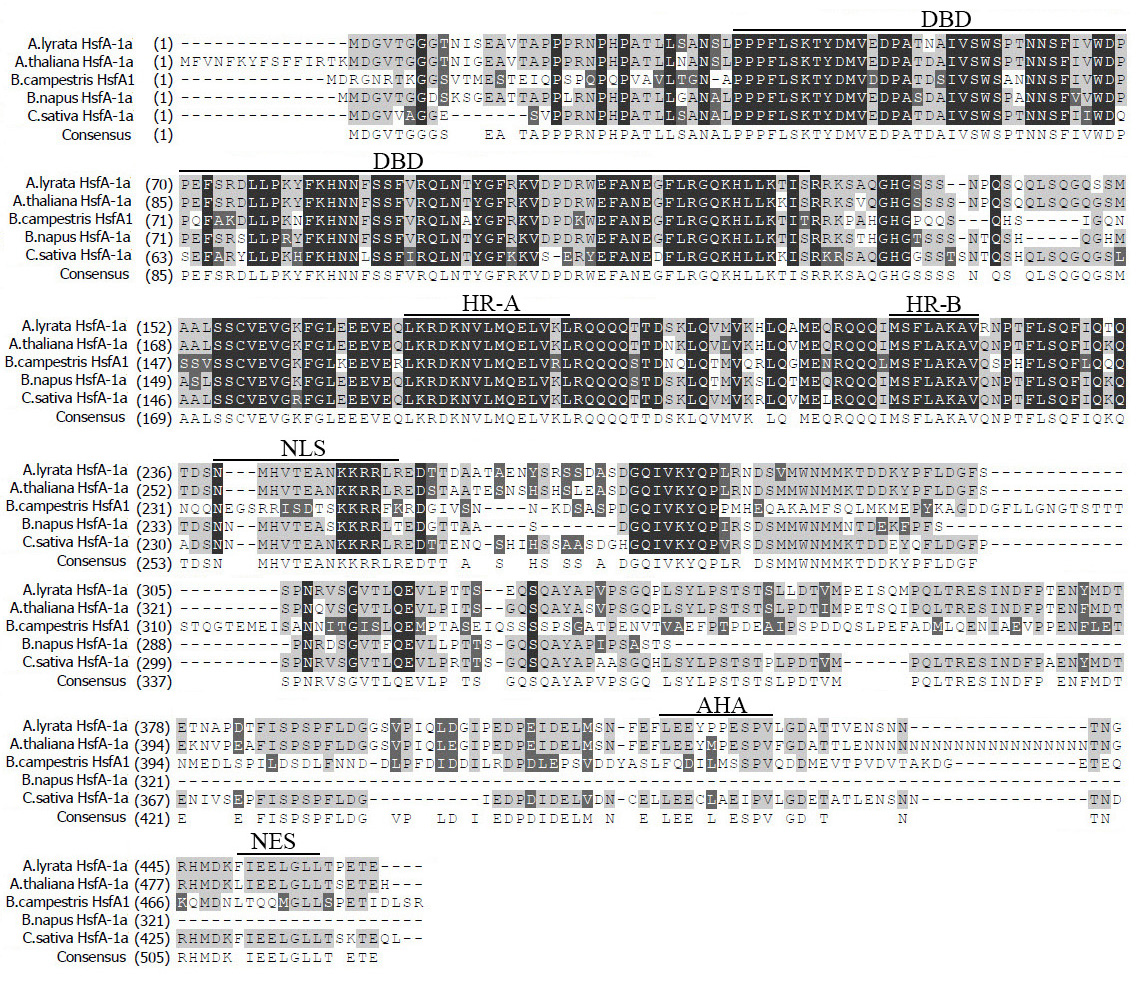

Supplement: S2 Fig — AtHsfA-1a (Arabidopsis thaliana, NCBI amino acid accession number NP_193510.1), AlHsfA-1a (Arabidopsis lyrata, XP_020873200.1), CsHsfA-1a (Camelina sativa, XP_010434623.1), BnHsfA-1a (Brassica napus, XP_013711617.1). DBD, DNA-binding domain; HR-A and HR-B, hydrophobic repeat regions A/B; NLS, nuclear localization signal; AHA, activator peptide motifs; NES, nuclear export signal. (JPG) [file pone.0207277.s002.jpg]

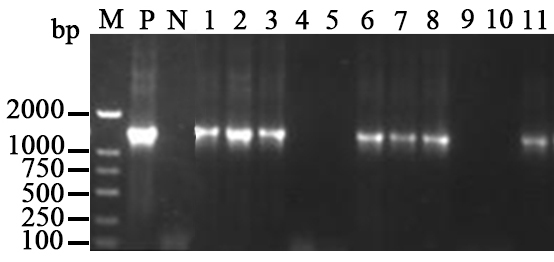

Supplement: S3 Fig — M, size marker; N, untransformed plant (negative control, N); P, p2300+-BcHsfA1 (positive control, P). (JPG) [file pone.0207277.s003.jpg]

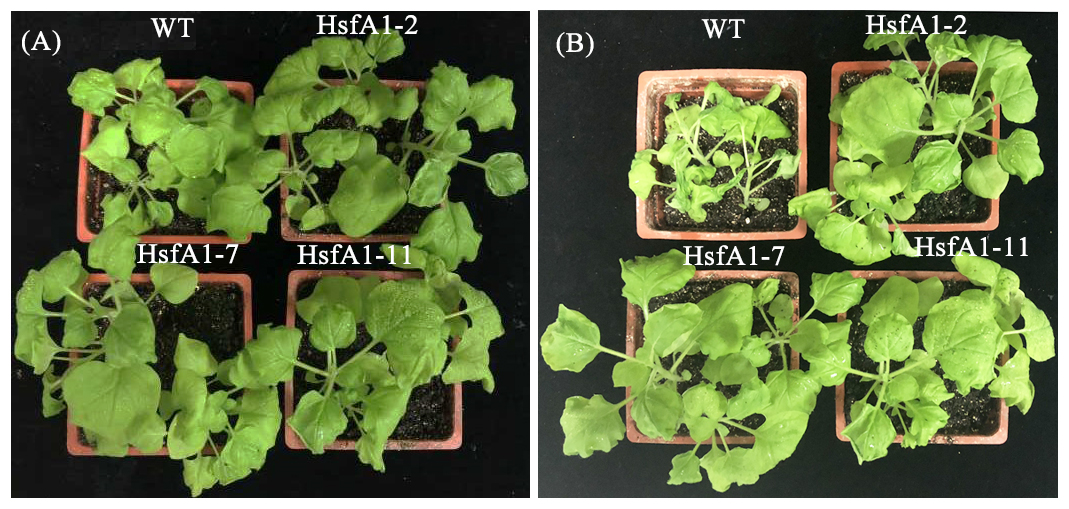

Supplement: S4 Fig — (JPG) [file pone.0207277.s004.jpg]
