# Supplementary material for: Overexpression of BcHsfA1 transcription factor from Brassica campestris improved heat tolerance of transgenic tobacco
Source: PLoS One. 2018 Nov 14;13(11):e0207277. doi: 10.1371/journal.pone.0207277 (PMC6235349; doi:10.1371/journal.pone.0207277)
Supplement: S1 Table — Primers used in this study. (DOCX) [file pone.0207277.s005.docx]

**Supplement Table S1** Primers used in this study

| **Primers** | **Sequences (5’-3’)** |
| --- | --- |
| BcHsfA1-ORF F | ATGGATCGGCGGTAACAGAAC |
| BcHsfA1-ORF R | TTACCTTGAAAGATCTATGGT |
| BcHsfA1-35S-F23 | GAGGACCTAACAGAACTCGCC |
| BcHsfA1-QR | CCGTCCTTCGTTGTAACATCCA |
| BcHsfA-real-F | CGGTTGATGATTACGCTT CACTCT |
| BcHsfA1-real-R | CCGTCCTTCGTTGTAACATCCA |
| NtSOD-real-F | CGGCAATTAGCGGTGACATA |
| NtSOD-real-R | ATGGCGTCATGTAGCTGTTC |
| NtPOD-real-F | CTCCATTTCCATGACTGCTTTG |
| NtPOD-real-R | GTTGGGTGGTGAGGTCTTT |
| NtLEA5-real-F | GTTACCATACCACGTCCCATAG |
| NtLEA5-real-R | GAGCTAGGACGCTCCATATTT |
| NtERD10C-real-F | AACGTGGAGGCTACAGATCG |
| NtERD10C-real-R | GTTCCTCTTGGGCATGAGTT |
| NtERD10D-real-F | GAGGACACGGCTGTACCAGT |
| NtERD10D-real-R | GCGCCACTTCCTCTGTCTT |
| NtHSP17.6-real-F | CTGATTCCGAGCTTCTTTGG |
| NtHSP17.6-real-R | CCAGGAAGATCCACCTTGAA |
| NtHSP18.2-real-F | TCGAGTGCTCGAATTGATTG |
| NtHSP18.2-real-R | TAAGGAACTTTCCGCTGCTC |
| NtHSP70-real-F | CTTAGAAGGTTGAGAACTG |
| NtHSP70-real-R | GGTAATGGTGGAGTAGAA |
| NtHSP82-real-F | TTACATTGGATGCTGAACA |
| NtHSP82-real-R | CTTACACAACAGGCTCAA |
| NtHSP90-real-F | TGAGACTGCCCTCCTCACCT |
| NtHSP90-real-R | ACCTCCTCCATCTTGCTACCC |
| NtHSP101-real-F | GGCGATAGATTGCACCAAAGA |
| NtHSP101-real-R | GCCCCAAGAAAAGGAATGAAC |
| NtActin-real-F | CTGCTGGAATTCACGAAACA |
| NtActin-real-R | GCCACCACCT TGATCTTCAT |
